# Does isolated atlantoaxial fusion result in better clinical outcome compared to occipitocervical fusion?

**DOI:** 10.1186/s13018-019-1525-y

**Published:** 2020-01-09

**Authors:** Katharina E. Wenning, Martin F. Hoffmann

**Affiliations:** Department of General and Trauma Surgery, BG University Hospital Bergmannsheil Bochum, Buerkle de la Camp-Platz 1, 44789 Bochum, Germany

**Keywords:** Occipitocervical fusion, Atlantoaxial fusion, Magerl-Gallie, Cervical spine, Injury, Outcome

## Abstract

**Background:**

The C0 to C2 region is the keystone for range of motion in the upper cervical spine. Posterior procedures usually include a fusion of at least one segment. Atlantoaxial fusion (AAF) only inhibits any motion in the C1/C2 segment whereas occipitocervical fusion (OCF) additionally interferes with the C0/C1 segment.

The purpose of our study was to investigate clinical outcome of patients that underwent OCF or AAF for upper cervical spine injuries.

**Methods:**

Over a 5-year period (2010–2015), consecutive patients with upper cervical spine disorders were retrospectively identified as having been treated with OCF or AAF. The Numeric Pain Rating Scale (NPRS) and the Neck Disability Index (NDI) were used to evaluate postoperative neck pain and health restrictions. Demographics, follow-up, and clinical outcome parameters were evaluated. Infection, hematoma, screw malpositioning, and deaths were used as complication variables. Follow-up was at least 6 months postoperatively.

**Results:**

Ninety-six patients (male = 42, female = 54) underwent stabilization of the upper cervical spine. OCF was performed in 44 patients (45.8%), and 52 patients (54.2%) were treated with AAF. Patients with OCF were diagnosed with more comorbidities (*p* = 0.01). Follow-up was shorter in the OCF group compared to the AAF group (6.3 months and 14.3 months; *p* = 0.01). No differences were found related to infection (OCF 4.5%; AAF 7.7%) and revision rate (OCF 13.6%; AAF 17.3%; *p* > 0.05). Regarding bother and disability, no differences were discovered utilizing the NDI score (AAF 21.4%; OCF 37.4%; *p* > 0.05). A reduction of disability measured by the NDI was observed with greater follow-up for all patients (*p* = 0.01).

**Conclusion:**

Theoretically, AAF provides greater range of motion by preserving the C0/C1 motion segment resulting in less disability. The current study did not show any significant differences regarding clinical outcome measured by the NDI compared to OCF. No differences were found regarding complication and infection rates in both groups. Both techniques provide a stable treatment with comparable clinical outcome.

## Background

The craniocervical junction represents a complex anatomical region consisting of two essential joints, the atlanto-occipital joint and the atlantoaxial joint [1]. Mobility of the cervical spine is complex and requires a combination of individual vertebral motion segments. The normal range of motion of the cervical spine contains six possible directions with the C0 to C2 region being the keystone for range of motion in the upper cervical spine. The atlantoaxial joint mostly accounts for rotation in the cervical spine. The rotational range of motion of a well-functioning C1/C2 segment was reported to be 23 to 39° [2], whereas rotation of the occiput on the atlas does not exist effectively due to the depth of the atlantal sockets. Its primary directions of motion are flexion and extension. The C0/C1 segment contributes 23 to 25° of flexion/extension of the skull, and the atlantoaxial motion segment adds an additional 10 to 22° [2–4].

Injuries to the upper cervical spine represent a serious entity including a wide spectrum of pathology ranging from benign to life threatening [1]. Especially in the elderly, these injuries are not uncommon and their incidence will continue to grow due to increasing life expectancy and higher activity levels of the elderly population [5–9]. Currently, the emphasis centers on surgical treatment to obtain good alignment and stability to allow early mobilization. Degenerative changes may compromise surgical stabilization. Therefore, various methods of fixation and/or fusion for upper cervical spine disorders have been described and successfully performed [10–12].

For posterior fixation of the upper cervical spine, two different categories of treatment options are available. Atlantoaxial fusion (AAF) procedures only interfere with the atlantoaxial motion segment (C1/C2). The AAF is the more demanding procedure compared to the occipitocervical fusion (OCF) but provides greater range of motion by preserving the C0/C1 motion segment. OCF leads to further and considerable limitation of movement compared to atlantoaxial fusion alone. After OCF, there is virtually no extension, flexion, and rotation in the upper cervical spine.

According to our literature research, no comparison of clinical outcome comparing occipitocervical fusion and C1/C2 fusion has been performed previously. Therefore, the aim of this study was to compare clinical outcomes of patients with a totally impaired range of motion at the upper spine (OCF) to patients with a preserved motion of the C0/C1 joint. According to our null hypothesis, OCF patients should have greater impairment regarding pain and everyday activities.

## Methods

### Patients

This study was an Institutional Review Board-approved retrospective and prospective cohort study of patients who underwent surgical treatment for fractures of the upper cervical spine between January 2010 and August 2015 in one referral trauma center. Surgeries were performed by four fellowship trained spine surgeons. The involved patients were retrospectively identified from the clinics database based on a computer query of Current Procedural Terminology (CPT) codes for fractures of the upper cervical spine. Informed consent was obtained, and patients were prospectively evaluated.

Inclusion criteria were age greater or equal to 18 years, injuries of the upper cervical spine (C0–C2), surgical treatment by AAF or OCF, no preoperative paraplegia or tetraplegia, signed patient information, and follow-up greater than 6 months. Exclusion criteria were additional anterior stabilization, metastatic disease or pre-existing infection, and insufficient medical record or radiographic data.

### Surgical techniques

#### Atlantoaxial fusion

**Fig. 1:**
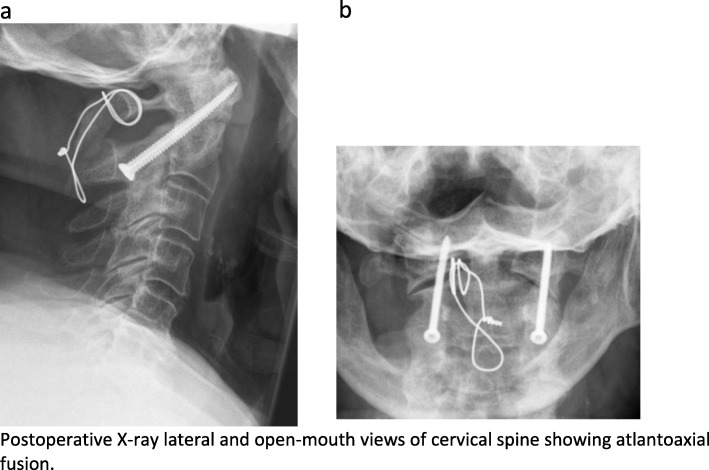
**a**, **b** Postoperative X-ray lateral and open-mouth views of the cervical spine showing atlantoaxial fusion

For AAF, the Magerl-Gallie technique was performed. Figure 1 a and b show the postoperative X-ray lateral and open-mouth views of AAF. All patients underwent general anesthesia and were positioned in a prone position utilizing a Mayfield clamp for reduction and fixation. For fixation of the C1/C2 complex, two transarticular screws and a C1/C2 cerclage with additional autologous bone grafting was performed. The placement of transarticular screws was similar in technical details to the technique described by Magerl et al. in 1979 [13]. Iliac crest bone graft harvested from the posterior iliac crest was utilized for posterior spondylodesis. The placement of the bone graft was between the posterior arch of C1 and the spinous process of the C2 vertebra. The lamina of the C2 vertebra and C1 arch were decorticated prior to the application of the H-shaped bone graft. To hold the graft in place, a sublaminar cerclage wire was passed beneath the arch of C1 and then wrapped around the spinous process of C2 (Gallie technique [14]). C1/C2 facet joints were curetted to enhance fusion.

#### Occipitocervical fusion

Occipitocervical fusion (OCF) was performed in a prone position with the patients’ head fixed with a Mayfield clamp similar to the atlantoaxial fusion under general anesthesia. For occipitocervical fixation, two different cervical spine system were utilized (VERTEX SELECT®, Medtronic Inc., Minneapolis, MN and NEON®, Ulrich Medical, Germany; respectively). Screws were inserted as lateral mass screws. Additional bone grafting was performed at the surgeon’s discretion.

#### Clinical assessment

For clinical outcome measurement, the Neck Disability Index (NDI) [15] and Numeric Pain Rating Scale (NPRS) were used to evaluate postoperative neck pain and health restrictions in daily routine. Therefore, the questionnaires were sent to all patients after at least 6 months by mail. The number and quality of returned questionnaires were assessed. Perioperative parameters such as fixation constructs, blood transfusions, and operation time were recorded and analyzed utilizing the hospital charts. Infection, postoperative hematoma, screw malpositioning, implant failure, neurologic disabilities, and deaths were utilized as complication variables.

#### Statistical analysis

For statistical analysis, Excel (Microsoft Excel for MAC, version 16.24) and SPSS version 23.0 (IBM, Chicago, IL) were utilized. To compare continuous variables (age, follow-up time, operation time, in-patient stay), a *T* test was performed. Fisher’s exact test was utilized to compare nominally scaled data. Significance was defined as *p* < 0.05. *p* values were rounded to one decimal place.

## Results

Between 2010 and 2015 106 (male: *n* = 47 (44.3%), female: *n* = 59 (55.7%)), patients were treated for upper cervical spine injuries. Thereof, 10 (9.4%) patients with preoperative tetraplegia (*n* = 8), paraplegia (*n* = 1), or brain damage (*n* = 1) were excluded. A total of 96 patients (male: *n* = 42 (43.8%), female: *n* = 54 (56.2%)) met the inclusion criteria. Of our patient population, 44 underwent occipitocervical fusion (OCF group) and 52 underwent transarticular atlantoaxial fusion in the Magerl-Gallie technique (AAF group) based on the surgeon preference. Patient age at the time of operation averaged 79 years (OCF group 78.0 ± 8.0 years versus 72.4 ± 10.1 years in the AAF group; *p* > 0.05).

All patients had a recent history of trauma. In all, the most common fracture was the odontoid process fracture (*n* = 88 (91.7%)). Of these 88 patients, 60 had isolated dens fracture, 7 had odontoid fracture combined with C2/C3 spondylolisthesis, and 21 had odontoid fracture with atlas fracture. Three patients sustained an isolated atlas fracture (Gehweiler type 3). The types of fracture are shown in Table 1*.*

**Table 1:** Fracture classification and types of fracture

Fracture classification		Number
Atlas fractures (all)		24
Gehweiler type 1		7
Gehweiler type 2		3
Gehweiler type 3		13
Gehweiler type 4		0
Gehweiler type 5		0
Unknown		1
Odontoid fractures (all)		88
Anderson/D’Alonzo type 1		0
Anderson/D’Alonzo type 2		69
Anderson/D’Alonzo type 3		13
Unknown		6
Traumatic spondylolisthesis of axis (all)		7
Hangman’s fracture		2
Unknown		5
Type of fracture	OCF	AAF
Atlas fractures*		
Gehweiler type 1	0	0
Gehweiler type 2	0	0
Gehweiler type 3	3	0
Gehweiler type 4	0	0
Gehweiler type 5	0	0
Odontoid fractures°		
Anderson/D’Alonzo type 1	0	0
Anderson/D’Alonzo type 2	32	37
Anderson/D’Alonzo type 3	4	9
Unknown	0	6
Combined fractures		
Odontoid fracture + atlas fracture	13	8
Odontoid fracture + traumatic Spondylolisthesis of axis	5	2

*Single injury

°Single injury and combined injuries

Follow-up averaged 10.4 months (6 months to 5.9 years). Follow-up of the OCF group was shorter compared to that of the AAF group (OCF 6.3 months versus AAF 14.3 months; *p* = 0.01). There was no significant difference between the two groups in terms of sex, age, types of fractures, time of hospital stay, and body mass index (BMI). Significantly, more OCF patients suffered from hypertension and coronary heart disease compared to the AAF group (*p* = 0.02). Baseline demographic and clinical data are shown in Table 2.

**Table 2:** Baseline demographic and clinical data of the patients

Characteristic	Value		
	AAF group	OCF group	*p*
Number of patients	52	44	
Sex (male/female)	29/23	19/25	
Average age (years)	72.4 ± 10.1	78.0 ± 8.0	
Average HS (days)	18.8 ± 26.5	16.1 ± 9.3	
BMI (kg/m^2^)	27.2 ± 3.9	24.7 ± 4.6	
FU (months)	14.3	6.3	0.01
Average OT (min)	139.8	136.3	
Blood transfusion (*n*)	78	57	
Average PL (NPRS)	2.0	2.9	
NDI score (%)	21.4	37.4	
Cervical spine system			
VERTEX SELECT® (*n*)		24	
NEON® (*n*)		20	
Extent of fixation			
C0–C3 (*n*)		12	
C0–C4 (*n*)		22	
C0–C5 (*n*)		7	
C0–C6 (*n*)		3	
Comorbidities			
Hypertension (*n*)	33	38	0.01
CHD (*n*)	13	21	0.01
Malignancy (*n*)	6	9	
T2DM (*n*)	4	6	
COPD (*n*)	3	5	
Anticoagulation (*n*)	21	22	
Complication			
Infection/hematoma (*n*)	4	2	
Screw dislocation (*n*)	5	1	
Persistent instability		2	
Infection rate (%)	7.7	4.5	
Revision rate (%)	17.3	13.6	
Death rate (*n*/%)	14/31.8	13/25	

*HS* hospital stay, *FU* follow-up, *OT* operation time, *PL* pain level, *NPRS* Numeric Pain Rating Scale, *COPD* chronic obstructive pulmonary disease, *CHD* coronary heart disease, *T2DM* type 2 diabetes mellitus

Surgery was successfully performed in 96 patients. The average operation time (defined as the time from incision to skin closure) was 136.3 ± 52.3 min in the OCF group versus 139.8 ± 31.9 min in the AAF group (*p* > 0.05).

**Fig. 2:**
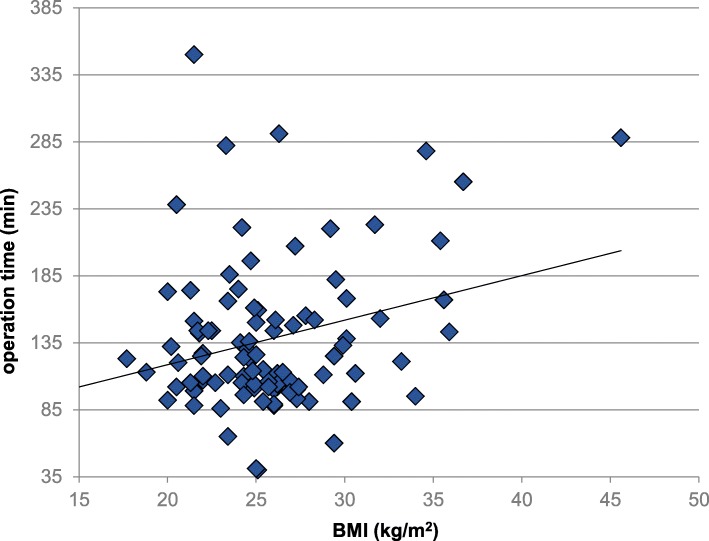
The correlation of BMI and the operation time for the patient population

Operative time was related to BMI for both surgical techniques (*p* = 0.01). Figure 2 presents the correlation of BMI and the operation time for our patient population. Transfusion rate did not differ between the OCF and AAF groups.

Postoperatively, there was no case of nerve damage or cerebrospinal fluid leakage. No soft tissue irritation or wound infections were observed at the donor sites of the autologous iliac bone graft. The most common postoperative complications encountered in our series included hematoma and/or infection in the affected surgical region (OCF, *n* = 2; AAF, *n* = 4). Postoperative computed tomography (CT) showed malpositioning of screws/rods in one patient with OCF and 5 patients with AAF. Two patients of the OCF group had a persistent instability of the upper cervical spine postoperatively and underwent a reversion surgery. One patient sustained a cardiopulmonary arrest intraoperatively due to suspected primary cardiac event. Therefore, he underwent surgery for OCF a few days later. The postoperative revision rate was 13.6% in the OCF group versus 17.3% in the AAF group (*p* > 0.05). No differences were found related to hematoma and/or infection rate (OCF 4.5%; AAF 7.7%).

Among the 96 patients, 27 patients died during the follow-up. There was no intraoperative death. Six patients (OCF, *n* = 1; AAF, *n* = 5) died during hospital stay: 4 patients died due to postoperative multiple organ failure, 1 patient died to a heart attack, and finally, 1 death happened due to pacemaker dissociation. There was no significant difference between the death rate of the OCF group compared to the AAF group (14 (31.8%) and 13 (25%), respectively).

**Fig. 3:**
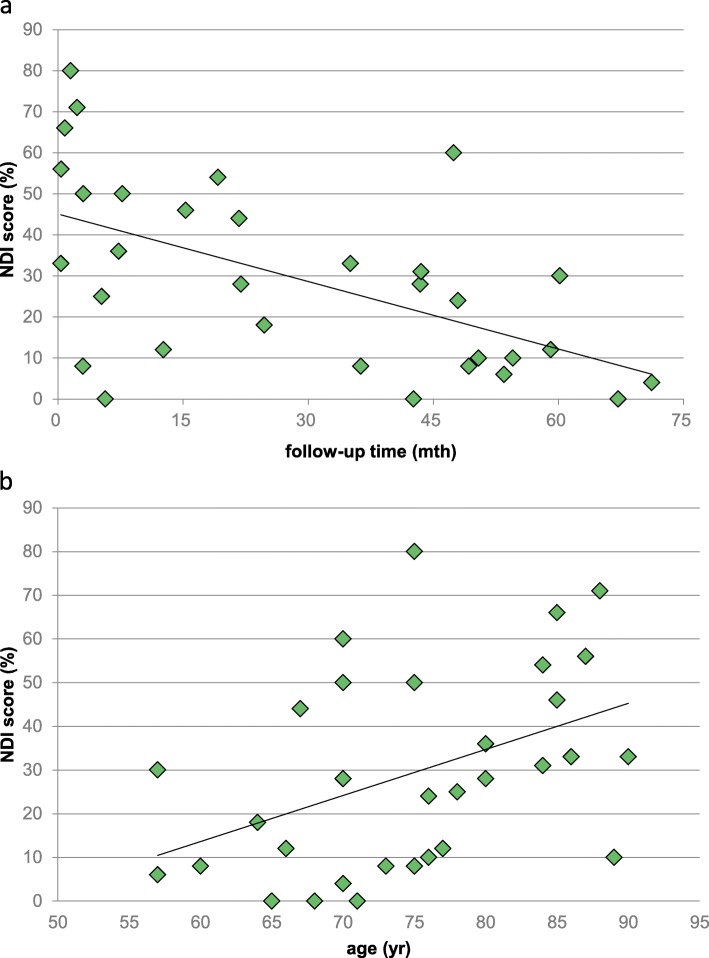
**a**, **b** The correlation between NDI score and follow-up time and between NDI score and age

No significant differences were discovered in both groups related to postoperative neck pain: pain reduction was rated sufficient and satisfying for all patients according to the NPRS. Patients who underwent OCF indicated average pain levels of 2.9. The mean NPRS score for the AAF group was 2.0. Regarding bother and disability in daily living, no differences were discovered utilizing the NDI score. The average NDI score of the OCF group was 37.4% compared to 21.4% in the AAF group. A significant reduction of disability in daily routine measured by the NDI was observed with greater follow-up for all patients (*p* = 0.01). Moreover, NDI scores increased with increasing age for both operations (*p* = 0.01). Figure 3 a and b illustrate the correlation between NDI score and follow-up time and between NDI score and age, respectively.

## Discussion

Preservation of mobility and physiological range of motion combined with stable fixation is one of the major goals in orthopedic trauma surgery. The complex area of the occiput, atlas, and axis achieves the greatest mobility of any segment within the spine [16]. The major part of the spine’s flexion, extension, and rotation occurs in the upper cervical spine (C0 to C2) [2, 3, 16, 17]. The primary motion of the occipitoatlantal segment is the flexion and extension, and additionally, the atlantoaxial joint is very mobile in axial rotation. As a result, the upper cervical spine is of utmost importance for mobility of the head. Therefore, it presents unique challenges to stable internal fixation in case of injuries. The complex anatomy of the cervical spine as well as the great range of motion in this area influences the surgeons regarding decision-making and execution of internal fixation. More stable procedures tend to reduce the range of motion significantly and, therefore, often result in greater impairment of the patients.

In most western countries, demographics show a graying trend over the last 30 years. The percentage of elderly patients with cervical spine injuries, secondary to falls, has been on the rise and will continue to increase in the future related to increased life expectancy. Geriatric patients have a higher risk of low-energy injuries secondary to osteopenia, osteoporosis, and decreased total mobility due to degenerative changes [18]. Improvements in health contributed to the growth of the older population over the past century and result in greater activity of the elderly population. Upper cervical spine disorders are severe injuries due to the risk of myelopathy and death from proximal spinal cord compression.

Management of cervical spine injury in the elderly remains controversial because of many influencing factors such as the quality of the bone, osteoarthritis, classification, and type of the fracture [6, 7, 10, 19, 20]. Treatment might be complicated by numerous comorbidities and reduced bone quality. Therefore, injuries to the C1/C2 region are often treated posteriorly by fusion procedures which might be advantageous in a current meta-analysis [21]. It can be difficult to achieve a balance between optimizing the stabilization and minimizing the impairment of motion.

Atlantoaxial fixation for C1/C2 injuries provides immediate biomechanical stability to the atlantoaxial complex and results in high arthrodesis rates (> 90%) [12, 22–26]. The advantage of this surgical technique is the greater range of motion by preserving the C0/C1 motion segment compared to occipitocervical fusion. Postoperatively, flexion and extension of the upper cervical spine are preserved. But atlantoaxial fixation is not always feasible in the elderly.

There are multiple OCF techniques currently available, and they have all proved high fusion rate and reduced pain levels [27, 28]. Currently, rod-wire systems, rigid rod-screw fixation, and occipital hooks and cervical claws are being used, and all shown to have high fusion rates (89–100%) [28–31]. Therefore, occipitocervical fusion allows a valid and reliable surgical technique in upper cervical spine disorders [11, 32–35] but is accompanied by severe limitation of range of motion of the neck [2, 36]. As a consequence, flexion and extension are reduced by 23–24.5°. Additionally, lateral bending and axial rotation are limited by 3.4–5.5° and 2.4–7.2°, respectively [2]. Normally, the complex C0/C1 joint allows for > 50% of all head and neck movements [37].

Despite distinct impairment of range of motion, OCF often is the first choice for craniocervical instability in the elderly [38, 39]. Several studies [40–43] have shown that as age increases cervical spine mobility decreases. In the geriatric population, there is a frequent presence of osteoarthrosis of the upper cervical spine with subsequent primary limitation of extension and flexion of the neck. Kuhlmann [40] worked out that the elderly had significantly less range of motion at the upper cervical spine than the younger control group. This motion loss was greater for cervical extension and least for cervical flexion or rotation. This leads to the conclusion that geriatric patients with age-related restrictions, especially with reduced extension, do not feel strongly impaired after OCF.

Cappuccio et al. [44] recommended OCF in case of post-traumatic cervical instability because the C0/C1 joint sacrifice in an elderly ankylotic spine does not make a relevant clinical difference in the final functional outcome. In case of C2 fractures, Shousha et al. [45] compared anterior odontoid screw fixation with AAF and also concluded that the posterior motion preservation techniques should be limited to younger patients.

In this study, comparisons were made between demographic data, clinical outcomes, and complications after OCF and AAF based on the data of patients with at least 6-month follow-up. Regarding clinical outcomes, no statistically significant differences, such as NPRS or NDI score, were found between OCF and AAF. Moreover, additional fusion of the C0/C1 segment, with lack of flexion, extension, and rotation of the neck (OCF group), did not lead to an increase in pain or disability in daily life. Postoperatively, both study groups presented with nearly the same pain level (OCF, mean NPRS score 2.9; AAF, mean NPRS score 2.0). There were no statistical differences of NDI scores between both groups, but patients who underwent OCF had a slightly higher NDI score (moderate pain level) compared to patients after AAF (mild pain level). Hu et al. [39] compared the clinical outcome parameters between OCF and AAF in treatment of the unstable atlas fracture and reported that all patients had a significant improvement of neck pain after fixation of the upper cervical spine. But there was a statistically significant difference in the satisfaction of these both groups (*p* = 0.0085). All OCF patients (*n* = 20) complained of severe restriction of cervical spine flexion, extension, and rotation, and only 14 patients were satisfied with their outcome. Both groups had a restricted rotation of the neck, yet the additional OCFs’ restriction of the extension and flexion led to significant self-reported disability. The average age of the OCF group was 53 years (35–78) [39], and therefore, these patients were relatively young and might have had higher expectations of their postoperatively function.

A significant improvement in neck pain was also documented by Hu et al. [46]. The average NPRS score of the AAF group was 1.0 ± 0.4 and 1.3 ± 0.9 of OCF patients postoperatively (*p* < 0.01). They used the Japanese Orthopaedic Association (JOA) score to assess the severity of clinical symptoms in their patients. According to our results, both groups presented with mild symptoms after fixation of their upper cervical spine. In summary, and according to other studies [28, 39, 47, 48], OCF and AAF enable a sufficient improvement of pain with a reasonable level of activities in daily life.

Our study shows a negative correlation between follow-up time and NDI score. The longer the follow-up time, the better the NDI score. Similar results were found by Yuan et al. [49]. This implies a kind of patients’ adaptation to their disability and health state. Additionally, a positive correlation between the patients’ age and NDI score was found in our actual study. Advanced age was related to increased postoperative disability, thus an increased NDI score. The slightly higher NDI score of our OCF group can be explained by the fact that this patient collective has significantly more comorbidities than the AAF patients and displays a shorter follow-up period (OCF group 6.3 months versus AAF group 14.3 months). It is therefore to be expected that the NDI score of the OCF group will be even better with an extended follow-up time. It can be assumed that the current, relatively slight difference between both groups will even more decline. With the reduction of disabilities during follow-up, further approximation of the NDI score of AAF and OCF patients might be expected.

Regarding postoperative adverse events, no statistically significant differences, such as infection or revision rate, were found between our study groups. The postoperative revision rate was 13.6–17.3%, and the hematoma and/or infection rate was 4.5–7.7%. In a systematic review, Winegar et al. [50] reported about 68 documented cases of postoperative adverse events such as wound complications. Of these 68 cases, 21 cases exhibited wound infection and dehiscence. Therefore, their postoperative infection rate (30.9%) was much higher than in our study groups. Additionally, a higher wound infection rate (13.3%) was reported by another study [51].

We acknowledge the limitations of our study. The major limitation of this study was its retrospective design and relatively small sample sizes. Due to the advanced age of the collective, many patients were deceased at time of follow-up. The lack of long-term follow-up data is another limitation of this study. Additionally, we did not have data on preoperative scores (NDI and NRPS) or range of motion. Therefore, further data and studies are warranted.

## Conclusion

Instability of the upper cervical spine endangers patients and neurologic integrity. Stable fixation techniques are essential tools in the treatment of these entities. Occipitocervical fusion and atlantoaxial fusion are both effective and safe treatment options for upper cervical spine disorders. In the hand of an experienced surgeon, both procedures provide safe stabilization for the upper cervical spine. Despite an additional fixation of the C0/C1 segment (OCF group) with a consecutively increased impairment of motion compared to the AAF group, both surgical methods lead to comparable clinical outcome in our elderly study population.

## Data Availability

The datasets used and/or analyzed during the current study are available from the corresponding author on reasonable request.

## Abbreviations

AAF: Atlantoaxial fusion
BMI: Body mass index
CPT: Current Procedural Terminology
JOA: Japanese Orthopaedic Association
NDI: Neck Disability Index
NPRS: Numeric Pain Rating Scale
OCF: Occipitocervical fusion
